# Quantum-like cognition and decision-making in the light of quantum measurement theory

**DOI:** 10.1098/rsta.2024.0372

**Published:** 2025-11-27

**Authors:** Miho Fuyama, Andrei Khrennikov, Masanao Ozawa

**Affiliations:** ^1^ College of Letters, Ritsumeikan Daigaku, Kyoto, Kyoto Prefecture, Japan; ^2^ Int. Center Math. Modeling in Physics and Cognitive Science, Karl Linnaeus University, VÄXJÖ, Sweden; ^3^ Center for Mathematical Science and Artificial Intelligence, Academy of Emerging Sciences, Chubu University, Kasugai, Aichi Prefecture, Japan; ^4^ Graduate School of Informatics, Nagoya University, Nagoya, Aichi Prefecture, Japan; ^5^ Innovation Design Office, RIKEN, Wako, Saitama Prefecture, Japan

**Keywords:** cognition, decision-making, quantum-like modelling, quantum measurement theory, question order and response replicability effects, sharp repeatable non-projective measurements

## Abstract

We characterize the class of quantum measurements that matches the applications of quantum theory to cognition (and decision-making)—quantum-like modelling. Projective measurements describe the canonical measurements of the basic observables of quantum physics. However, the combinations of the basic cognitive effects, such as the question order and response replicability effects (RREs), cannot be described by projective measurements. We motivate the use of the special class of quantum measurements, namely, *sharp repeatable non-projective measurements*—$SR\bar{P}.$ This class is practically unused in quantum physics. Thus, physics and cognition explore different parts of quantum measurement theory. Quantum-like modelling is not the automatic borrowing of the quantum formalism. Exploring the class $SR\bar{P}$ highlights the role of *non-commutativity of the state-update maps generated by measurement back action*. Thus, ‘non-classicality’ in quantum physics as well as quantum-like modelling for cognition is based on two different types of non-commutativity, of operators (observables) and instruments (state-update maps): *observable non-commutativity* versus *state-update-non-commutativity*. We speculate that distinguishing quantum-like properties of the cognitive effects is the expression of the latter, or possibly both.

This article is part of the theme issue ‘Quantum theory and topology in models of decision making (Part 1)’.

## 1. Introduction

The quantum information revolution, which is often called the second quantum revolution, stimulated not only the development of quantum technologies but also applications of the formalism of quantum theory outside of physics, especially to studies on cognition, consciousness and decision-making*—quantum-like modelling*.^1^ Cognitive tasks, e.g. questions asked to humans, are realized as measurements, and quantum measurement theory plays the crucial role in such studies. In fact, this theory is more complex than it is often presented in textbooks. Typically (starting from von Neumann [10]), quantum measurements are described as projective ones.^2^ The class P of projective measurements covers the measurements of the basic quantum physical observables.^3^ The class P is also important for applications to cognition and decision-making. The first stage of the development of quantum-like modelling was characterized by the employment of the projective measurements [14,15]. However, later on, it becomes clear that the class P does not cover all cognitive effects [16]. We note that, although P is widely explored in quantum mechanics, this is only a special class of quantum measurements.

This is a good place to make the following terminological remark. We distinguish the notions ‘measurement’ and ‘observable’.

Measurement is characterized by a pair of its statistical properties: the ‘outcome probability map’ describing the probability distribution of its outcome for any input state and the ‘state-update map’ describing the state change resulting from measurement. Here, we only consider real-valued measurements, the outcomes of which are represented as real numbers. In quantum theory, the outcome probability map is represented by a positive (or probability) operator valued measure (POVM). Thus, any measurement is represented by two components, the POVM and the state-update map. In quantum theory, they are unified in the sole notion of ‘quantum instrument’ [11,12,17–23]. Thus, we shall use the terms quantum measurement and instrument interchangeably.^4^

To see this briefly, let H be the complex Hilbert space describing a quantum system S; for simplicity, we work in finite-dimensional spaces. Any state of the system S is represented by a density operator ρ. The space of linear operators on H is denoted by L(H); the space of density operators (i.e. positive operators with unit trace) is denoted by D(H). A linear operator acting in L(H) is called a *superoperator*. Consider a measurement, or more concretely, a *measuring apparatus* A(x) with an *output variable* x taking values x in the real line ℝ. Let the outcome probability map be defined as


(1.1) ρ↦Pr{x=x‖ρ},

which maps the initial state ρ (the state just before the measurement) to the probability Pr{x=x‖ρ} of obtaining the outcome x=x in the initial state ρ. Similarly, let the state-update map be


(1.2) $$\rho \mapsto \rho_{\left\{\text{x}=x\right\}},$$

which maps the initial state ρ to the updated state (the state just after the measurement) $\rho_{\left\{\text{x}=x\right\}}$ given the outcome x=x. The ‘instrument’ I associated with the apparatus A(x) is defined by


(1.3) $$I(x)\rho =\mathrm{Pr}\{\text{x}=x\|\rho \}\rho_{\left\{\text{x}=x\right\}},$$

for any ρ∈D(H). Then, as $\text{Tr}[\rho_{\left\{x=x\right\}}]=1$, we obtain


(1.4)Pr{x=x‖ρ}=Tr[I(x)ρ],(1.5)ρ{x=x}=I(x)ρTr[I(x)ρ].

Thus, the instrument I integrates both the outcome probability map ρ↦Pr{x=x‖ρ} and the state-update map ρ↦Pr{x=x‖ρ} in a single mathematical object. Now, it can be shown that the mapping ρ↦I(x)ρ defined in equation (1.3) is naturally extended to a positive superoperator on L(H) for every x∈X, where $X=\{x_{1},...,x_{n}\}\subseteq \mathbb{R}$ is a set of possible outcomes. Such that $\sum_{x\in X}I(x)$ is trace-preserving, and these properties mathematically characterize the notion of instruments as originally introduced by Daview & Lewis [17]; see [23] for details.

In contrast, ‘observable’ generally means a physical quantity that can be measured or observed, where a physical property is included as a {0,1}-valued quantity. In quantum formalism, observables of the system are represented by self-adjoint operators on the ‘state space’ (the Hilbert space of state vectors) of the system, and every observable takes its value as one of its eigenvalues in any state in the corresponding eigensubspace. The projections on to the eigensubspaces are called the ‘spectral measure of the observable’, which is a projection-valued (or projective) POVM, and conversely every projective POVM is associated to a unique observable in this way, according to the spectral theory for self-adjoint operators. The probability of the outcome from the measurement of an observable in a given state is determined by the spectral measure of the observable using the Born formula. In contrast, a non-observable physical quantity has no corresponding self-adjoint operator. For example, the one-dimensional quantum harmonic oscillator has the Hilbert space $L^{2}(R)$ on which there are self-adjoint operators corresponding to position, momentum and energy, but there is no self-adjoint operator corresponding to phase. Thus, for the one-dimensional quantum harmonic oscillator, position, momentum and energy are observables, but phase is not an observable [24].

Measurement and observables are coupled here. A measurement is called a ‘measurement of an observable’ if its POVM is identical to the spectral measure of the observable. Thus, different measurements with the same projective POVM are considered to describe different ways (with different state-update maps) of measuring the same observable corresponding to that POVM. One of such measurements is the ‘projective measurement’ of that observable, the state-update map of which projects the input state on to the eigensubspace corresponding to the outcome.

Any measurement with a non-projective POVM is considered as a measurement of some observable with some error. The uncertainty principle applies to any measurement to give a quantitative relation between the error of measuring one observable versus the disturbance in another observable; the universally valid form of the error–disturbance relation has been studied extensively for the last two decades [22,25–30].

**Figure 1.**
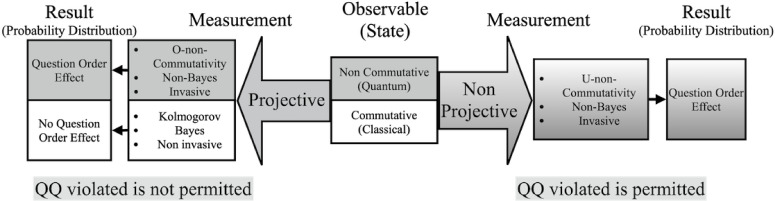
Schematic presentation of the structure of measurement theory, classical versus quantum: (see subsection (a) for detailed explanations and decoding of its blocks).

In addition to the one-to-one correspondence between the observables and the projective POVM, general POVMs are often considered as generalized observables, especially in quantum information theory. Such generalized observables can also be useful in quantum-like modelling. However, structuring the discussion in the form ‘observables versus POVMs’ is the wrong strategy, since such a discussion would not highlight the structure of state-update maps. From our viewpoint, the essence of the difference between physical and cognitive measurements is precisely in this structure. The situation is complicated, and its complexity will be illuminated in this paper (see the scheme in figure 1).

Our main message (to the quantum-like community as well as to quantum physicists who may become interested in new applications) is that to model cognition, one should use a special part of quantum measurement theory—*sharp repeatable non-projective measurements* (the meaning of each term will be explained in §4). Denote this class of measurements by the symbol $SR\bar{P}.$ Such measurements are practically unused in quantum physics, although they are covered by the general quantum measurement theory that studies all the physically realizable measurements. It seems that *physics (as described by conventional textbooks) and cognition are basically coupled to two different classes of quantum measurements,* namely, P and $SR\bar{P}.$ (By ‘basically’ we mean that their characteristic effects are described within these classes.^5^)

This search for a proper class of measurements for the quantum-like framework for cognition and decision-making leads to ‘reincarnation’ of the old debate on the quantum–classical interplay. It seems that going outside of purely physical applications leads to a novel view on this interplay. The employment of the class $SR\bar{P}$ highlights the role of non-commutativity of the state-update maps generated by measurement back action formulated within quantum instrument theory. Thus, non-classical effects are related to two different types of non-commutativity, of operators (observables) and instruments (state-update maps). So, we should distinguish two types of non-commutativity, *observable non-commutativity* (O-non-commutativity) and state*-update-non-commutativity* (U-non-commutativity).

The order effect in quantum measurements is expressed as the U-non-commutativity. In quantum mechanics, it is well known that the U-non-commutativity follows from the O-non-commutativity according to the uncertainty principle. However, in quantum-like modelling of cognitive effects, the U-non-commutativity without the O-non-commutativity plays an important role in quantum-like modelling of cognition and decision-making. We speculate that distinguishing quantum-like cognitive effects is the expressions of U-non-commutativity. This does not exclude that O-non-commutativity also plays the important (but not crucial!) role in quantum cognition.

In the Wang–Busemeyer model [14], the order effect follows from the O-non-commutativity of the projections representing a pair of questions, whereas in the Ozawa–Khrennikov model [31], the order effect does not follow from the O-non-commutativity, since the pair of questions is represented by two commuting projections. In both models, the order effect is formulated as U-non-commutativity of the belief-update map. In Kolmogorov’s classical probability theory, all observables (i.e. classical random variables) commute and the belief-update maps are Bayesian, or equivalently non-invasive, so they do not show the U-non-commutativity. Thus, modelling the question order effect (QOE) needs non-Bayesian, or non-invasive, belief-updates maps.

Our suggestion to identify advanced cognitive measurements as belonging to class $SR\bar{P}$ is based on quantum-like modelling of the combinations of a few cognitive effects within quantum measurement theory; see articles [31–33] on combining the question order and response replicability effects (RREs; §3). These cognitive effects involve sufficiently advanced cognition, using memory (at least short-term) and the conscious state of mind. Our aim is to characterize such cognitive measurements. Less advanced cognitive measurements, e.g. as in psychophysics, need not belong to $SR\bar{P}$ (nor P).

In addition, we point to the role of the QQ equality (§3, equality (3.1)) in characterization of the class of advanced cognitive measurements. In article [14], this equality was derived for P measurements, and it was demonstrated that it holds for plenty of data from social opinion polls [15]. However, recently, it was found that some data on aesthetic perception in the literature show the violation of the QQ inequality [34]. Hence, such data cannot be described within the class projective measurements P. At the same time, measurements from the class $SR\bar{P}$ can both satisfy and violate this equality.

We briefly discuss the issue of (non-)invasiveness of measurements. Invasiveness is equivalent to the possibility of violating the formula of total probability (FTP) and to non-Bayesian update of probabilities. A consistent discussion on this issue is possible within the von Neumann algebra framework—‘non-commutative probability theory’; within it, one can compare the quantum and classical measurement theories based, respectively, on the state spaces consisting of density operators and probability measures [35]. We just point out that quantum observables can be measured only invasively (except for trivial observables corresponding to constant quantities). We stress that the violation of the FTP (and the employing non-Bayesian update) is the probabilistic expression of the disjunction effect (DE) [4,7].

Preparation of the concrete mental states is a complex problem. Therefore, in quantum-like modelling, working within the state-independent formalism is not so natural. It may be useful to work with state-dependent statements, say, instead of considering non-commuting operators, [A,B]≠0, to consider operators non-commuting in the concrete state ρ [33]. We shortly discuss the state-dependent approach in §5c; we also mention state-dependent non-invasiveness (validity of the FTP for some density operator ρ) in §2.

The paper is of a conceptual nature and also contains a short review of the mathematical basis of quantum measurement theory (§2). The review is brief, but goes deeply into the advanced part of this theory, the calculus of quantum instruments.

### (a) Detailed explanations and decoding of the blocks of the schematic representation

At the centre of figure 1, the algebraic structure of the observable quantities (so-called observables, physical quantities, random variables) of the system to be measured is classified based on observable non-commutativity or observable commutativity. Observable non-commutativity leads to quantum probability theory, while observable commutativity leads to classical probability theory. The left-hand arrow from the central box indicates the role of projective measurements in both quantum and classical theories. The upper section of the next box explains that in quantum theory, projective measurements demonstrate O-non-commutativity (i.e. the non-commutativity of state-update maps that is derived from observable non-commutativity), describe invasive measurements and lead to non-Bayesian state updates (or belief updates, if the state represents a belief rather than the object’s state). In this context, the state update is also known as wave packet collapse in quantum mechanics. The von Neumann–Lüders projection postulate in the conventional formulation of quantum mechanics requires that every measurement of an observable be a projective measurement. The lower section of the next box explains that in classical theory, projective measurements describe non-invasive measurements and lead to the Bayesian state updates (belief updates). The Kolmogorov probability theory postulates that every measurement of a random variable should be projective when defining the sequential joint probability distribution of arbitrary random variables, from which the Bayesian update rule (from the prior probability to the posterior probability) follows. The leftmost box explains that projective measurements of any pair of questions represented by non-commuting projections in quantum theory exhibit the QOE, as used in the Wang–Busemeyer model [14], whereas projective measurements of any pair of questions in classical theory do not exhibit this effect. The right-hand arrow from the central box indicates the role of non-projective measurements in both quantum and classical theories. The next box explains that non-projective measurements demonstrate U-non-commutativity (i.e. the non-commutativity of state-update maps not necessarily derived from observable non-commutativity), describe invasive measurements and lead to non-Bayesian state updates (belief updates). The rightmost box explains that non-projective measurements of a pair of questions represented by projections, whether or not they are non-commuting, show the QOE. The QOE caused by non-projective measurements of a pair of questions represented by commuting projections is shown to be compatible with the RRE in the Ozawa–Khrennikov model [31,32]. The leftmost bottom line explains that a pair of projective measurements of two projections (questions) shows the QQ equality, while the rightmost bottom line explains that a pair of non-projective measurements does not necessarily show this equality.

## 2. Mathematical formalism of quantum measurement theory

A projective measurement is described by a *projection-valued measure*. Let $X=\{x_{1},...,x_{n}\}\subseteq \mathbb{R}$ be a set of possible outcomes, and let $x\to E_{A}(x)$ be a map from X to projections such that $E_{A}(x)E_{A}(y)=\delta_{x,y}E_{A}(x)$ and $\sum_{x}E_{A}(x)=I.$ This map determines the projection-valued measure defined on the subsets Δ of X as $\mu_{A}(\Delta )=\sum_{x\in \Delta }E_{A}(x).$ For the quantum state given by the density operator ρ, the probability of the outcome A=x is given by the Born rule $P(A=x|\rho )=\text{Tr}\rho E_{A}(x),$ and the measurement with the outcome A=x induces the state update


(2.1) $$\begin{array}{lrr} & \rho \mapsto \rho_{x}=\frac{E_{A}(x)\rho E_{A}(x)}{\text{Tr}[E_{A}(x)\rho E_{A}(x)]}. & \end{array}$$

Since $E_{A}(x)E_{A}(x)=E_{A}(x),$ this measurement is repeatable. The self-adjoint operator A representing the observable A to be measured by this projective measurement is given by the spectral decomposition $A=\sum_{j=1}^{n}x_{j}E_{A}(x_{j})$; the set X is the spectrum (the set of eigenvalues) of A, and $E_{A}(x_{j})$ is the projection operator on to the eigensubspace of A corresponding to the eigenvalue $x_{j}$.

Consider a measurement with outcome (variable) A taking the discrete range of values $X=\{x_{1},...,x_{m},...\}.$ Any map $x\to I_{A}(x),$ where for each x∈X, the map $I_{A}(x)$ is a positive superoperator and $I_{A}(X)=\sum_{x}I_{A}(x):D(H)\to D(H),$ or equivalently $I_{A}(X)$ is trace-preserving, is called a *quantum instrument*^6^ with outcome A. The probability for the outcome A=x is given by the generalization of the Born rule


(2.2) $$P_{\rho }(A=x)=\text{Tr}\;[I_{A}(x)\rho ].$$

We note that a measurement with the outcome A=x generates the state update


(2.3) $$\rho \to \rho_{x}=\frac{I_{A}(x)\rho }{\text{Tr}[I_{A}(x)\rho ]}.$$

Conditional probability P(B=y|A=x‖ρ) is defined as


(2.4) $$P(B=y|A=x\|\rho )=P(B=y\|\rho_{A=x})=\frac{\text{Tr}[I_{B}(y)I_{A}(x)\rho ]}{\text{Tr}[I_{A}(x)\rho ]}.$$

By using the quantum conditional probability, we define sequential joint probability distribution,


(2.5) $$P(A=x,B=y\|\rho )=P(A=x\|\;\rho )P(B=y|A=x\|\rho )=\text{Tr}[I_{B}(y)I_{A}(x)\rho ].$$

Repeatability of measurement is described as P(A=x|A=x‖ρ)=1 or P(A=x,A=x‖ρ)=P(A=x‖ρ). This is A−A replicability in RRE; A−B−A and B−A−B replicability are defined as


(2.6) $$\begin{array}{rl} P(A=x,B=y,A=x\|\rho ) & =P(A=x,B=y\|\rho ),\\ P(B=x,A=y,B=x\|\rho ) & =P(B=x,A=y\|\rho ). \end{array}$$

It is crucial that the same observable A can be measured by a variety of instruments generating the same probability distribution, but different state updates. Each quantum instrument determines POVM given by $\Pi (x)=I^{*}(x)I.$ Operators Π(x),x∈X are called *effects*; they are positive semi-definite self-adjoint and sum to the unit operator: $\sum_{x\in X}\Pi (x)=I.$ An instrument is sharp if its POVM is of the projective type, i.e. all effects are mutually orthogonal projections. An instrument (measurement) belongs to the class P if its POVM is of the projective type and the same projections determine the state update, that is, $I_{A}(x)\rho =E_{A}(x)\rho E_{A}(x).$ An instrument belonging to the class $SR\bar{P}$ has the projective POVM, but the state-update map is not of the projective type.

### (a) Non-invasiveness versus invasiveness

Let $I_{A}=(I_{A}(x))$ be a quantum instrument with a set of outcomes $X=\{x_{1},...,x_{m}\};$ $I_{A}$ is called non-invasive if $I_{A}(X)=\text{id}$, where id is an identity superoperator. Hence, for any density operator ρ,$\sum_{x\in X}I_{A}(x)\rho =\rho ,$ or $\sum_{x\in X}p(A=x\|\rho )\rho_{A=x}=\rho .$ Instrument $I_{A}$ is called invasive if $I_{A}(X)\neq \text{id}.$

Let $I_{A}$ be a non-invasive instrument, and let $I_{B}$ be an arbitrary instrument. Then,


(2.7) $$\text{Tr}[I_{B}(y)\rho ]=\sum_{x\in X}p(A=x\|\rho )\text{Tr}[I_{B}(y)\rho_{A=x}].$$

Thus, the FTP of classical probability theory holds:


(2.8) $$p(B=y\|\rho )=\sum_{x\in X}p(A=x\|\rho )p(B=y|A=x\|\rho ).$$

The correct framework for analysis of the interplay between non-invasive and invasive instruments is the von Neumann algebra framework—‘non-commutative probability theory’. In standard quantum formalism, the notion of non-invasiveness is trivialized by the following statement. A quantum instrument $I_{A}$ is non-invasive if $I_{A}(x)=k_{x}\text{id},$ where $k_{x}\geq 0,\sum_{x}k_{x}=1.$ Here, it is natural to introduce the notion of the state-dependent non-invasiveness. An instrument $I_{A}$ is non-invasive in the state ρ if $I_{A}(X)\rho =\rho .$ This implies the validity of the FTP (equation (2.8)) for any instrument $I_{B}.$

## 3. Disjunction, question order and response replicability effects and the QQ equality

In this section, we briefly review some cognitive effects that might be considered as signatures of ‘U-non-commutativity’ (see §5 for the discussion of the meaning of this term).

DE is the violation of the Savage Sure Thing Principle. Mathematically, it is described as the violation of the FTP (see equation (2.8); see [4,7,36] for its quantum-like modelling).

QOE expresses dependence of the sequential joint probability distribution of answers to questions A and B on their order, that is, $p_{AB}≠p_{BA}$ (see equation (2.5) for the definition of sequential joint probability distribution). This effect is well confirmed experimentally in cognitive psychology and sociology, e.g. [37]. Its quantum-like modelling was performed by Wang & Busemeyer [14] with projective measurements.

RRE is expressed as follows. Alice can be asked two questions A and B in different orders. Suppose that first she was asked the A-question and answered it, e.g. with ‘yes’: Then Alice is asked the B-question and gives an answer to it. Then she is asked the A-question again; typically, Alice would repeat with probability one her original answer to the A-question, ‘yes’. This effect is A−B−A response replicability. In the absence of the intermediate B-question, this is A−A replicability, or called *repeatability* in quantum theory. Combination of A−B−A and B−A−B replicability forms RRE (see equation (2.6)).

Why is it so important to consider the combination of QOE and RRE? Their coexistence shows that QOE is present in the normal experimental condition, or shows that QOE is not a result of our irrationality or our lack of adequate memory. Under the condition that people can make consistent judgements using adequate memory, they show QOE.

We remark that in psychology and sociology, DE and QOE were well studied, both theoretically and experimentally [37]. In contrast, RRE have not yet been well studied. This effect attracted the attention of psychologists in connection with quantum-like modelling [16]. Up to now, only one experiment was performed [38], while the interpretational issue was the subject of hot debates (see comments to this article on the Public Library of Science One webpage; see also [39]). The authors of that article claimed that their experiment showed a violation of RRE, and the debate was concentrated on the validity of such an interpretation for the experiment’s output. However, the possibility of not violating RRE was the seed of interest in it within quantum-like modelling of cognition [16,31–33]. In contrast, the heuristically evident presence of this effect in decision-making and its possible combination with QOE disturbs the quantum-like project for cognition. As was shown in article [16], the combination QOE + RRE cannot be modelled with P measurements. The experiment reported in article [38] cannot help with the resolution of this problem. Hence, one should recognize that the class P is not sufficiently wide to cover all cognitive effects and their combinations. In [32], it was suggested to leave this class and explore the measurements of the class $SR\bar{P}.$

As was found by Wang & Busemeyer, using plenty of data collected in social opinion polls constrained by a special equality that they called *the QQ equality* [14]:


(3.1) $$\begin{array}{lrlr} & q & =p(ByAy)+p(BnAn)-[p(AyBy)+p(AnBn)] & \\ & & =p(AyBn)+p(AnBy)-[p(ByAn)+p(BnAy)]=0, & \end{array}$$

where Ay,By and An,Bn denote the answers ‘yes’ and ‘no’ to the questions A and B. This equality can be derived for quantum measurements belonging to the class P [14]. In fact, this is one of the tests for the applicability of P measurements. *If experimental data do not satisfy the QQ equality, then it cannot be described with such measurements*. We point out that such data were collected in the experiment reported in article [34].

In [14], this equality was discussed in connection with QOE. Quantities $q_{y}=p(ByAy)-p(AyBy)$ and $q_{n}=p(BnAn)-p(AnBn)$ characterize the degree of QOE. Their equality can be treated as a special quantum-like constraint. In the Wang–Busemeyer model, QOE was coupled to non-commutativity of the operators A and B representing the questions A and B and hence incompatibility of these questions, where two observables or questions are said to be *incompatible* if the corresponding operators do not commute. However, in articles [31,32], it was shown that QOE can be modelled by a pair of instruments measuring a pair of commuting observables represented by projection operators that have non-projective state-update maps and at the same time satisfy the QQ equality.

## 4. Sharp repeatable non-projective invasive measurements

All quantum measurements are mathematically described as POVMs, in the sense that every quantum measurement has a unique POVM that describes the probability distribution of the outcome of the measurement. Typically, measurements with projective POVMs are referred to as sharp measurements, and measurements with non-projective ones are referred to as unsharp.

Incompatibility of observables A and B means that they cannot be measured simultaneously with complete accuracies for both observables. The Heisenberg uncertainty principle is typically treated as representing the trade-off between the accuracies (or errors) of measurements of these observables. However, Heisenberg’s original trade-off relation [40] has been shown not to be universally valid, and a universally valid reformulation has been first derived by Ozawa [22,27]. Eventually, the violation of the original relation and validity of the new relation have been experimentally observed for certain pairs of observables [26,41,42].

To be more precise, for a pair of conjugate observables Q and P, Heisenberg’s original relation [40] is of the form


(4.1) $$\begin{array}{lrr} & \epsilon (Q)\epsilon (P)\geq \hbar /2 & \end{array}$$

for their ‘root-mean-square errors’^7^ ϵ(Q) and ϵ(P) with Planck’s constant ℏ divided by 2π, and Ozawa’s relation is of the form


(4.2) ϵ(Q)ϵ(P)+ϵ(Q)σ(P)+σ(Q)ϵ(P)≥ℏ/2,

with s.d. σ(Q),σ(P) in the initial state. Kennard [43] derived another relation


(4.3) $$\begin{array}{lrr} & \sigma (Q)\sigma (P)\geq \hbar /2 & \end{array}$$

for s.d. σ(Q) and σ(P), which is universally valid, but not relevant to the limitation to measurement accuracies in any way. Those relations are extended to an arbitrary pair of observables A,B by replacing the lower bound ℏ/2 by |⟨AB−BA⟩|/2 introduced by Robertson [44] to generalize Kennard’s relation (equation (4.3)), where ⟨⋯⟩ stands for the expectation value in the initial state.

We speculate that sharpness is one of the distinguishing features of advanced cognitive measurements, e.g. answering a question involves conscious information processing and leads to definite answers, say ‘yes’–‘no’. As was mentioned, the psychophysical tasks can be described by unsharp measurements. Moreover, as was mentioned, data collected in aesthetic perception in literature violate the QQ equality [34]. Such a violation can be easily modelled with unsharp measurements [45]. However, we want to emphasize that violation of the QQ equality can be modelled even with sharp measurements.

Sharpness and unsharpness only concern the POVMs of measurements. Now we turn to measurements with their state-update maps. (This is a good place to point out once again that the notion of POVM does not assume any concrete way of the state update.) We now proceed to the next level of measurements’ classification.

The sharp measurements are classified as repeatable and non-repeatable. Denote the class of sharp repeatable measurements as SR. Here, ‘repeatability’ means that if the first A measurement gives the outcome A=x, then the successive A measurement (performed immediately after the first one) gives the same outcome A=x with probability one. (This is A−A repeatability part of RRE.)

We remark that unsharp POVMs are not relevant, since they do not show repeatability in the finite-dimensional case [11, theorem 6.5]. The latter is another motivation to describe advanced cognitive tasks as sharp observables; otherwise, they cannot be coupled to repeatable measurements. Repeatability is an important feature of advanced cognition characterized by the employment of short-term memory. Once again, in psychophysics, repeatability can be violated. We note that projective measurements are repeatable: P⊂SR. But, SR≠P, there exist *sharp repeatable non-projective measurements*, and they are explored for application in cognitive psychology [31–33]. The class of such instruments, denoted as $SR\bar{P}$, seems to be the proper class for modelling advanced cognitive measurements.

Now we discuss the notions of *non-invasive* versus *invasive* measurement. A measurement is non-invasive if, for any state ρ, conditioning on measurement’s outcomes can be described by the classical FTP; otherwise, a measurement is invasive (see §2 for details). We recall that violation of the FTP can be interpreted as probabilistic interference [4–7]. Hence, invasive measurements can generate constructive and destructive interference of probabilities, and the non-invasive cannot. In applications to cognitive psychology, the violation of the FTP corresponds to DE. We remark again that the consistent analysis of the interplay between non-invasiveness and invasiveness can be performed within the von Neumann algebra framework—‘non-commutative probability theory’. We emphasize that all quantum measurements, in addition to trivial state updates, are invasive.

Let S be a cognitive system, e.g. a human. We summarize our reasoning for exploring $SR\bar{P}$ measurement:

— Sharp: S performs the accurate decisions of the ‘yes’–‘no’ type.
— Repeatable: S uses the short-term memory to be able to repeat the answer to a question A immediately after answering it.
— Non-projective: S’s memory is stable to perturbations generated by answering to other questions (RRE).
— Invasive: S makes probabilistic judgements involving interference of probabilities, violating the FTP and using a non-Bayesian probability update (DE).

We point out again that $SR\bar{P}$ corresponds to advanced cognition, including conscious information processing.

## 5. Quantumness: non-commutativity of observables versus state-update maps

### (a) P-class: non-commutativity ∼ non-commutativity of observables

Since the work of Heisenberg [46] (written around 100 years ago) that led to the employment of the matrix and later operator calculus [10] in quantum mechanics, *non-commutativity of the operators* representing observables has been considered as the main distinguishing feature of the mathematical formalism of quantum theory. Generally speaking, non-commutativity has been identified with the mathematical description of quantumness. This viewpoint is strongly supported by *the Heisenberg uncertainty relation*, e.g. in the form of the Schrödinger or more generally Robertson inequality. In turn, this relation led Bohr to the formulation of *the complementarity principle* [47,48], the basic methodological principle of quantum theory. Non-commutativity of operators is rigidly coupled to incompatibility of the corresponding observables, that is, the impossibility of their joint measurement, or, in other words, observables A and B are jointly measurable if and only if the corresponding self-adjoint operators A and B commute, [A,B]=0. An algebra of mutually commuting observables is considered as a classical structure within quantum theory. Such observables can be jointly measured, and they have the joint probability distribution determined by their joint projection-valued measure. In this sense, quantum physics can be considered as an extension of classical physics. This is a good place to mention the Koopman–von Neumann representation of classical phase space mechanics in that the classical position and momentum variables are described as commuting self-adjoint operators (cf., however, with [49]). This line of thinking on ‘quantumness’ is common in physics (see also [50] for quantum-like theory).

### (b) $SR\bar{P}$-class: non-classicality ∼ non-commutativity of state-update maps

As was discussed, the applications of the quantum formalism outside of physics, e.g. to cognition and decision-making, stimulate careful analysis of the interrelation of the notions ‘quantumness’ and ‘classicality’. Modelling of the combination of QOE and RRE stimulated us to go outside of the class P and work with the measurements of the class $SR\bar{P}.$ The corresponding observables used in this modelling [31–33] commute! From the traditional viewpoint, such models should be treated as classical. However, reproduction of QOE with $SR\bar{P}$ measurements prevents one from such a conclusion. Heuristically, one understands that some sort of non-commutativity should be involved. This is the non-commutativity of state-update maps,


(5.1) $$[I_{A}(x),I_{B}(y)]\neq 0,$$

for two quantum instruments $I_{A}$ and $I_{B}$ generating QOE.

In fact, we say that *instruments* $I_{A}$ *and* $I_{B}$ *show the QOE*, if


(5.2) $$\begin{array}{lrr} & P(A=x,B=y\|\rho )≠P(B=y,A=x\|\rho ) & \end{array}$$

for some x,yρ. Then, this is equivalent to the relation


(5.3) $$\text{Tr}[[I_{A}(x),I_{B}(y)]\rho ]\neq 0.$$

Thus, equation (5.1) is a sufficient condition for $I_{A}$ and $I_{B}$ to show QOE. From this viewpoint, instruments belonging to the class $SR\bar{P}$ are non-classical. At the same time, the operators A and B representing the observables in the quantum-like model for QOE + RRE must commute, [A,B]=0. The latter means that the observables can be jointly measurable, that they are compatible. From this viewpoint, they should be treated as classical ones.

Hence, there are two forms of non-classicality coupled to two forms of non-commutativity, for observables and state-update maps. We can speak about *observables non-commutativity* (O-non-commutativity) and *update non-commutativity* (U-non-commutativity). (We point out that for projective instruments, these two forms of non-commutativity are equivalent, O-non-commutativity ∼U-non-commutativity.) As was highlighted, such classifications of non-commutativity were stimulated by the development of quantum-like modelling. In physics, the P-class plays the crucial role, and, although generalized observables in the form of POVMs are actively used in quantum information theory, one typically does not understand that they are just derivatives of quantum instruments.^8^

It seems that the role of the non-commutativity condition (5.1) for quantum foundations was highlighted only recently [31–33]. The order effect is not of high interest in physics. The most important thing is that quantum physical systems do not exhibit RRE. Thus, the split of ‘non-commutativity’ into two counterparts is the new foundational problem that came to scientists’ attention through quantum-like studies.

In the original quantum-like study (P-model) [14,15], QOE was interpreted as the expression of non-commutativity of operators A and B representing the questions A and B; that is, incompatibility of observables, the impossibility to determine their joint probability distribution. In this framework, ‘non-commutativity of cognition’ is interpreted as O-non-commutativity, i.e. as in physics. Our $SR\bar{P}$ model showed that, generally such coupling to incompatibility of questions (tasks) is not supported by quantum measurement theory. Observables can have the joint probability distribution and, nevertheless, show QOE, since the sequential joint probability distribution of the answers to questions A and B differs from the joint probability distribution for the simultaneous measurement of A and B owing to the invasiveness of measurement; otherwise, QOE does not present.

This situation has the following cognitive interpretation. At each moment, a cognitive system S (as a human being) performing advanced cognitive tasks involving memory (at least short-term) has a consistent probabilistic picture for possible answers to the questions A and B. One might say that the $SR\bar{P}$ model recovers *mental realism*. In less advanced processing of cognitive information, S can operate within P-framework by demonstrating QOE and violating RRE.

### (c) State dependence of non-commutativity and classicality

As was emphasized in [33], the notions of non-commutativity and classicality are state-dependent. First, we look at O-non-commutativity. Consider observables with self-adjoint operators $A_{1},A_{2}$ and spectral measures $E_{A_{i}}(x),i=1,2.$ If for some state, e.g. a pure state |ψ⟩,$[E_{A_{1}}(x),E_{A_{2}}(y)]|\psi \rangle =0$ for all x,y, then the joint probability distribution is well defined,


p(A1=x1,A2=x2‖ψ)=‖EA1(x1)EA2(x2)ψ‖2=‖EA2(x2)EA1(x1)ψ‖2(5.4)=‖EA1(x1)∧EA2(x2)ψ‖2,

where ∧ stands for the infimum of two projections. Thus, for the state |ψ⟩, $A_{1}$ and $A_{2}$ can be treated as classical observables. But, for another state |ϕ⟩ such that $[E_{A_{1}}(x),E_{A_{2}}(y)]|\phi \rangle ≠0$ for some x,y, observables $A_{1}$ and $A_{2}$ behave as quantumly.

For the equivalence of state-dependent commutativity and the existence of the joint probability distribution for a family $A_{1},...,A_{n}$ of observables, we have the following theorem [52, theorem 5.2]: A family $A_{1},...,A_{n}$ of observables has its joint probability distribution $p(A_{1}=x_{1},\ldots ,A_{n}=x_{n}\|\psi )$ in a state |ϕ⟩, i.e.


(5.5) $$\begin{array}{rl} p(A_{1}=x_{1},\ldots ,A_{n}=x_{n}\|\psi )= & ||E_{A_{1}}(x_{1})\cdots E_{A_{n}}(x_{n})\psi |{|}^{2}\\ = & ||E_{A_{1}}(x_{1})\wedge \cdots \wedge E_{A_{n}}(x_{n})\psi |{|}^{2}, \end{array}$$

if and only if $[f(A_{1},...,A_{n}),g(A_{1},...,A_{n})]|\psi \rangle =0$ for any polynomials $f(A_{1},...,A_{n})$ and $g(A_{1},...,A_{n})$.

Consideration of such state-dependent compatibility of observables is especially important in quantum-like modelling of cognition, where preparation of a cognitive system S in an arbitrary state is practically impossible. Hence, state-independent compatibility versus incompatibility is practically impossible to check.

One can consider a weaker condition of state-dependent commutativity [33]:


(5.6) $$\begin{array}{lcr} & \langle \psi |[A_{1},A_{2}]|\psi \rangle =0. & \end{array}$$

It also implies certain classical features of observables (see [33]).

Now we turn to U-non-commutativity. Consider two instruments $I_{1}$ and $I_{2}$ such that


(5.7) $$[I_{1}(x),I_{2}(y)]\rho =0,$$

for all pairs (x,y). Then such instruments do not generate QOE for the state ρ, and from this viewpoint, they are classical in this state (even if the corresponding observables are projective and do not commute).

## 6. Concluding remarks

This note contributes to the foundational analysis of quantum-like modelling, a variety of applications of quantum theory outside of physics, e.g. to cognition and decision-making. The experience of the quantum-like studies shows that one cannot simply borrow the quantum formalism and apply it to model cognitive effects. The lesson of the attempts to apply it to describe the combinations of a few basic effects in cognitive psychology is that the class of projective measurements P is not wide enough for such a purpose [16] (cf. [14,15]). At the same time, this class covers the basic variables of quantum physics. Thus, it seems that physics and cognition are based on different parts of quantum measurement theory. Our studies on combination QOE + RRE [31–33] motivate us to shape the quantum-like cognition within the class $SR\bar{P},$ sharp repeatable non-projective measurements. This class is not well explored in quantum physics. Hence, quantum physics and quantum-like cognition (decision-making) are basically coupled to different domains of quantum measurement theory.^9^ We relate the class $SR\bar{P}$ to advanced cognition involving memory; we speculate that humans’ consciousness operates with such measurements. Less advanced mental processing might be described by measurements that do not belong to $SR\bar{P},$ in particular, by projective measurements.

As was shown in [31], the combination QOE + RRE and the QQ equality can be portrayed within $SR\bar{P},$ but generally $SR\bar{P}$ measurements can violate the QQ equality. The statistical data collected in the recent experiment on aesthetic perception in literature violate this equality [34]. Hence, it cannot be modelled within P. The QQ equality (as well as RRE) can be used as the complementary test to determine whether QOE can be portrayed with the projective measurements. The measurement basis for aesthetic perception should be analysed in more detail. It is not clear whether the class $SR\bar{P}$ is proper for such perception. Perhaps even the condition of sharpness is too strong. (We remark that instruments with unsharp POVMs can violate the QQ equality [45].)

This paper is devoted to the analysis of the applicability of quantum measurement theory to cognition and decision-making. Similar analysis is required for other areas of quantum-like modelling, e.g. finances [53–55].

## Data Availability

This article has no additional data.

## Footnotes

^1^
In this note, we cannot reflect even the basic publications in this area. A Google search generates 213 000 000 results for ‘quantum-like modelling’. We just mentioned a few monographs [1–8] and a review [9].

^2^
We highlight that the notion of a projective measurement means not only that the probability distribution of its outcomes is based on the projection-valued measure (spectral measure of a self-adjoint operator), but also that the measurement back action updates the original state by the projection on to the corresponding eigensubspace—the von Neumann–Lüders *projection postulate*.

^3^
Projective measurements, or more generally, accurate repeatable measurements, are possible only for observables with a discrete spectrum in the standard formulation of quantum mechanics [11,12], while they are possible within arbitrarily small errors [10,13].

^4^
The tricky point is that in the projective measurement, the state update is described by the same spectral projection-valued measure as the probability distribution. One might miss that this projection-valued measure carries two totally different meanings. To be rigorous, one should speak about projective instruments, not just projective observables.

^5^
Note that in quantum physics, non-repeatable measurements (the class $\bar{R}$) are also practically used for some basic observables, such as photon counting measurements of the photon number observable.

^6^
This is the very general definition of a quantum instrument given by Davies & Lewis [17,18]. To make quantum instruments physically realizable, i.e. described on the basis of unitary interaction between a system S and measurement apparatus M, the class of quantum instruments should be restricted. The proper class— completely positive instruments—was found by Ozawa [11].

^7^
Heisenberg originally called ϵ(Q) ‘die Genauigkeit, mit der der Wert Q bekannt ist’ (the accuracy with which the value Q is known) or ‘etwa der mittlere Fehler von Q’ (approximately the mean error of Q) but did not give a precise definition. A precise definition of ‘quantum root-mean-square error’ ϵ(A) for an arbitrary measurement of an observable A satisfying the requirements of soundness (if the measurement is accurate, then ϵ(A)=0) and completeness (if ϵ(A)=0 then the measurement is accurate) is given in [29]. A practical but not necessarily complete definition of ϵ(A) is given as the ‘root-mean-square’ of error operator M(τ)−A(0), where in the Heisenberg picture for the measuring process, A(0) is the measured observable A just before the measurement, and M(τ) is the meterobservable just after the measurement. In contrast, the s.d. σ(A) in the initial state is the ‘root-mean-square’ of A(0)−⟨A(0)⟩, which is independent of the measuring process and irrelevant to the measurement error or accuracy.

^8^
A recent paper [51] introduced a notion of observability for generalized observables (or POVMs) called ‘value reproducible measurability’ and proved that every sharp observable is value reproducibly measurable, but every unsharp generalized observable is not value reproducibly measurable. Thus, the observable status of unsharp generalized observables is questionable.

^9^
This is a good place to repeat once again that the notion of measurement is mathematically formalized on the basis of quantum instrument calculus. An instrument unifies two objects: a POVM that characterizes the probability distribution of outcomes and the state-update map that characterizes the measurement back action. Hence, attempts to perform the foundational analysis of quantum-like modelling by using just the notion of observable or POVM are misleading; in particular, it is misleading to reduce this analysis to the interplay of projective POVMs (observables) versus general POVMs.
